# RNA Stability: A Review of the Role of Structural Features and Environmental Conditions

**DOI:** 10.3390/molecules29245978

**Published:** 2024-12-18

**Authors:** Igor V. Kornienko, Olga Yu. Aramova, Anna A. Tishchenko, Dmitriy V. Rudoy, Michael Leonidas Chikindas

**Affiliations:** 1 Center for Agrobiotechnology, Don State Technical University, Gagarina Sq. 1, Rostov-on-Don 344003, Russia; ikornienko@yandex.ru (I.V.K.); dmitriyrudoi@gmail.com (D.V.R.); micromike123@yahoo.com (M.L.C.); 2 Federal Research Centre Southern Scientific Centre of the Russian Academy of Sciences, Chekhov Ave. 41, Rostov-on-Don 344006, Russia; 3 Department of Genetics Academy of Biology and Biotechnology, Southern Federal University, Stachki Ave. 194/1, Rostov-on-Don 344090, Russia; 4 Department of Big Data and Machine Learning, St. Petersburg National Research University of Information Technologies, Mechanics and Optics, Kronverksky Pr. 49, St. Petersburg 197101, Russia; tischenko.anna.a@yandex.ru; 5 Health Promoting Naturals Laboratory, School of Environmental and Biological Sciences, Rutgers State University, 65 Dudley Road, New Brunswick, NJ 08901-8525, USA; 6 Department of General Hygiene, I.M. Sechenov First Moscow State Medical University, Trubetskaya Str. 8, Bldg 2, Moscow 119048, Russia

**Keywords:** RNA, RNA stability, temperature, acidity of the medium, ionic composition, oxidative stress, methylation, RNA structure, RNA–protein interactions

## Abstract

The stability of RNA is a critical factor in determining its functionality and degradation in the cell. In recent years, it has been shown that the stability of RNA depends on a complex interaction of external and internal factors. External conditions, such as temperature fluctuations, the level of acidity of the environment, the presence of various substances and ions, as well as the effects of oxidative stress, can change the structure of RNA and affect its stability. Internal factors, including the specific structural features of RNA and its interactions with protein molecules, also have a significant impact on the regulation of the stability of these molecules. In this article, we review the main factors influencing RNA stability, since understanding the factors influencing this extremely complex process is important not only for understanding the regulation of expression at the RNA level but also for developing new methods for isolating and stabilizing RNA in preparation for creating biobanks of genetic material. We reviewed a modern solution to this problem and formulated basic recommendations for RNA storage aimed at minimizing degradation and damage to the molecule.

## 1. Introduction

The study of RNA stability is an urgent task of molecular biology, since RNA molecules are highly sensitive to various environmental factors, which makes it difficult to store and use them for research and medical purposes [1]. Since the 1970s, the scientific community has actively studied the mechanisms of RNA degradation and protection, including ribonuclease inhibition and chemical modifications of the molecule. Since then, RNA stability studies have become a critical area in molecular biology. Despite significant progress, RNA stabilization remains a challenging task in the context of the growth of RNAi technologies, such as mRNA vaccines, the development of therapeutics using noncoding RNAs, and biobanking. The aim of this article is to review the main factors affecting RNA stability and describe the existing approaches to its stabilization, providing science-based recommendations for the storage and handling of RNA samples.

In this article, we analyze in detail the stability of different types of RNA and discuss recent advances in the development of stabilization methods, such as chemical modifications of nitrogenous bases, the protection of RNA secondary and tertiary structures from degradation, and novel approaches to the use of stabilizing agents and proteins. Understanding these aspects of RNA stability is fundamental to the development of storage technologies and applications in biomedicine and biotechnology, especially for purposes such as vaccine development and the development of new therapeutic approaches.

## 2. Influence of RNA Structure on the Stability of the Molecule

### 2.1. RNA Primary Structure: Features and Chemical Modifications

The configuration of ribonucleic acid (RNA) is a critical factor that determines its functional characteristics as well as its ability for molecular interaction. The primary structure of RNA, which is a sequence of nucleotides, plays a key role in its stability and function [2]. 

**Figure 1:**
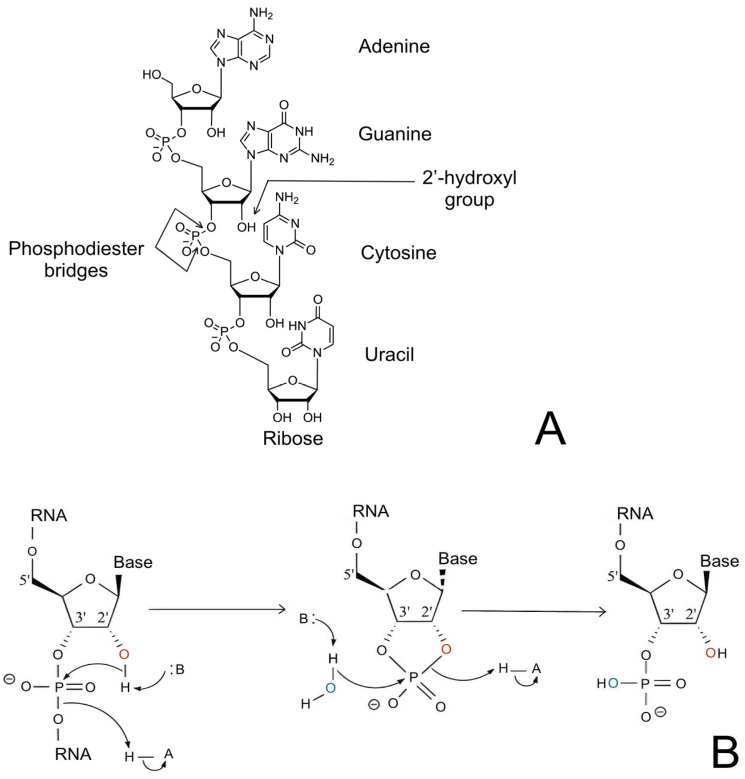
Structure and hydrolysis of RNA. (**A**) Chemical structure of RNA. (**B**) Mechanism of hydrolysis of phosphodiester bonds [4].

A key role in the structural instability of RNA is played by ribose, which contains an additional (as opposed to deoxyribose) hydroxyl group (OH-) at the 2′-position position (Figure 1A). This OH-group is able to chemically attack the neighboring phosphodiester bond, promoting its cleavage of the phosphodiester bond, especially in an alkaline environment (Figure 1B) [3].

Lindahl et al. pointed out that phosphodiester bonds in DNA are 200 times more stable than similar bonds in RNA (at neutral pH and physiological levels of Mg^2+^) [5]. The instability of phosphodiester bonds in RNA compared to DNA represents an important evolutionary advantage, allowing the cell to rapidly and efficiently regulate gene expression through rapid synthesis and the degradation of mRNA in response to changes in the external environment, which is critical for adaptation [6]. This mechanism also minimizes the risk of mutation accumulation in mRNA, preventing potential negative consequences for cellular function [7]. In addition, less stable RNA molecules are energetically more favorable for temporal functions, making them ideal for the dynamic control of cellular processes [8].

RNA hydrolysis is facilitated by divalent cations, like Ca^2+^ and transition metal ions, which act as catalysts [9]. At physiological pH values (around 7.4), the hydroxyl group of ribose at the 2′-position can act as a nucleophile by attacking the phosphorus atom of the phosphodiester bond connecting this nucleotide to the nearest nucleotide at the 3′- or 5′-end. This intramolecular reaction leads to the formation of a 2′,3′-cyclic phosphate intermediate and a 5′-hydroxyl group on the neighboring nucleotide [10,11].

The cyclic 2′,3′-phosphate can be further hydrolyzed depending on the reaction pathway [12]. The hydrolysis reaction results in the formation of 2′-phosphate and 3′-phosphate, which leads to the final breakage of the phosphodiester bond.

It is important to note that this process is highly dependent on the specific ionic environment and the presence of metal ions that can stabilize the transition state and promote catalysis. RNA hydrolysis is also affected by pH, with slightly alkaline conditions (e.g., pH 8.0) significantly accelerating the reaction due to the increased availability of hydroxide ions, which act as a base to activate the 2′-hydroxyl group [13].

The length and composition of the poly(A)-tail affect RNA stability [14]. Short poly(A)-tails (<50 nucleotides) cannot effectively protect the 3′-end of RNA from exonucleases. Long poly(A)-tails of >150 nucleotides can make RNA more resistant to degradation but in turn can prevent binding to ribosomes, thereby reducing the efficiency of RNA translation.

**Figure 2:**
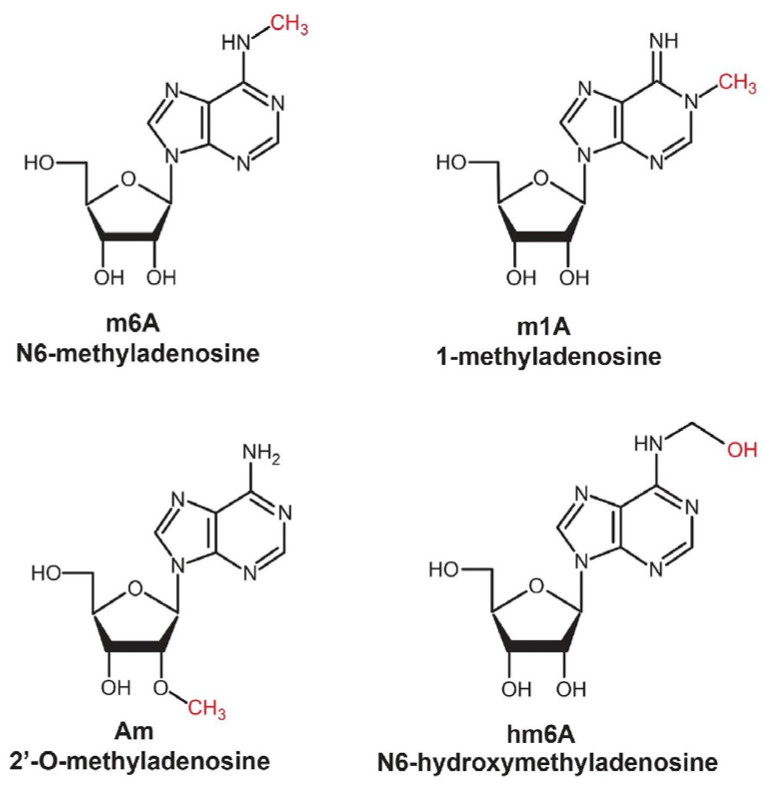
Chemical modifications of the poly(A) tail of RNA.

The composition of the poly(A) tail also affects RNA stability. The methylation of adenosine N6 (m^6A) in the poly(A) tail can compromise RNA stability, because methyl groups promote binding to poly(A)-binding proteins [15,16]. In contrast, hydroxymethylation of the poly(A) tail may possibly increase its stability, since hydroxyl groups are able to form additional bonds with poly(A)-binding proteins. The chemical modifications of the poly(A) tail of RNA are shown in Figure 2 [17].

Chemical modifications of RNA regulate its stability by influencing its structure, function, and protein interactions. Some strengthen the RNA molecule, while others accelerate its breakdown. One of the important mechanisms of such modifications is the change in the electrostatic charge of RNA, which reduces the ability of exonucleases to effectively bind to the molecule [18].

Moreover, nucleotide base modifications can enhance the interaction with RNA-binding proteins, such as PABPs, which not only protect RNA from degradation but also reduce the availability of sites for exonuclease binding by changing the conformation of the molecule [19].

Methylation is an important stabilizing factor of RNA: the addition of a methyl group (-CH_3_) to certain nucleotide positions protects it from degradation by preventing hydrolysis of phosphodiester bonds and regulating translation. The most studied modification is N6-methyladenosine (m^6A), which is added to the amino group of adenosine at the N6 position and reduces the susceptibility of RNA to degradation by blocking the binding of RNases. m^6A is involved in the regulation of mRNA stability and translation, making it an important factor in controlling gene expression [15,16]. This type of methylation reduces the free energy of stem-loop formation and helps stabilize the RNA secondary structure. Studies show that including m6A in the sequence can reduce the free energy of hairpin formation by ~2–4 kcal/mol, which prevents the unfolding of the structure and makes it more difficult for RNases to access [20].

Another type, 5-methylcytosine (m^5C), stabilizes RNA structure, protects it from degradation, and promotes mRNA export from the nucleus [21]. m5C methylation stabilizes RNA structures through internal base binding, which also reduces free energy by about 1–3 kcal/mol depending on the position of the modification and the overall sequence context. Simulations created with ViennaRNA demonstrate that m5C methylation leads to stem-loop stabilization in rRNA and tRNA, where it increases the strength of interactions and prevents degradation [20,22].

Other types of methylation also play key roles. The 2′-O-methylation of ribose (Nm) stabilizes the molecule, protecting it from hydrolysis [23]; 2′-O-methylation strengthens local interactions in RNA, reducing the flexibility of the sugar–phosphate backbone and making the structure less mobile. This reduces the likelihood of denaturation and increases the resistance of RNA to enzymatic hydrolysis and chemical attack, such as alkalis and RNases that cleave nucleotide bonds. Additionally, 2′-O-methylation improves the thermodynamic stability of RNA by reducing the free energy by an order of magnitude of ~0.5–1 kcal/mol per methylated nucleotide. The effect of Nm-modification varies depending on the specific sequence context and RNA structure, allowing the stability of individual elements, such as stems, hairpins, and UTR regions, to be regulated [24,25].

N1-methyladenosine (m^1A) is a modification of adenosine in which a methyl group is added to the N1 atomic position of the purine ring and promotes proper tRNA stacking and enhances its functionality [26]. Studies have shown that the presence of m^1A stabilizes tRNA, reducing free energy by ~0.7–1.5 kcal/mol depending on methylation position, which promotes more stable stacking and increases resistance to denaturation. Studies have shown that the presence of m^1A stabilizes tRNA, reducing free energy by ~0.7–1.5 kcal/mol depending on methylation position, which promotes more stable stacking and increases resistance to denaturation [20,27].

The methylation of N7-guanosine (m^7G) is a modification in which a methyl group (-CH_3_) is added to the nitrogen at the N7 position of guanosine. This modification creates a “cap” structure (m^7^G-cap) of the mRNA at the 5′-end and protects the molecule from 5′-exonucleases and maintains the stability of the mRNA [28]. Although the specific free energy reduction values for m^7G may vary depending on the sequence and RNA type, studies show that m^7G stabilizes RNA structure by strengthening hydrogen bonds and reducing the flexibility of the sugar–phosphate backbone, making the structure more compact and stable [29].

N6,2′-O-dimethyladenosine (m^6Am), present at the 5′-cap, further regulates mRNA stability and participates in its translation [30]. Thus, methylation is an important element of post-transcriptional regulation, ensuring RNA stability and functionality in the cell.

Pseudouridylation also plays an important role in stabilizing RNA by changing the structure of RNA and protecting it from degradation. An example of this is the pseudouridylation of uracil at the 55th position of tRNA in eukaryotes, which contributes to its stability. 

Uracilylation, which is the addition of uracil to the 3′-end of RNA, makes RNA more accessible to degradation enzymes, as is the case with eukaryotic mRNA [31].

In addition to these modifications, RNA stability can be affected by damage caused by reactive oxygen species. For example, the oxidative damage of guanine leads to the destabilization of RNA and makes it more susceptible to the action of RNases [32].

### 2.2. Features of RNA Secondary Structure

The secondary structure of RNA is a two-dimensional configuration resulting from interactions between nucleotide sequences and serves as the basis for the formation of the complex three-dimensional structure of the molecule. The secondary structure plays a key role in gene regulation, maintaining RNA stability and its ability to interact with proteins. The secondary structure is characteristic of tRNAs, rRNAs, microRNAs, and small interfering RNAs, fulfilling important functions in the cell. It is important to note that, although mRNA is mostly linear, some of its regions can form local secondary structures, such as hairpins, loops, and pseudoknots. Studies show that most eukaryotic mRNAs have an order of 10–15% of nucleotides involved in the secondary structure (loops, hairpins, etc.); although, some viral mRNAs may have regions forming diverse and denser secondary and tertiary structures, often exceeding 30–40% [33,34,35]. The secondary structure of mRNA also plays an important role in the stability of the molecule. For example, viral mRNAs often possess complex secondary structures that help them avoid the host immune response and efficiently utilize cellular mechanisms for viral protein synthesis [36].

The 3′-UTR region of the mRNA of the *TNF-α* (tumor necrosis factor alpha) gene contains AU-rich elements that form secondary structures [37]. Some mRNAs contain secondary structures that affect alternative splicing, resulting in the formation of different protein isoforms. For example, the mRNA of the gene *Fas*, encoding a receptor involved in apoptosis, forms a secondary structure in the intron before exon 6. This structure can influence the inclusion or exclusion of exon 6 in the final mRNA, which determines whether the resulting protein will be a membrane receptor that triggers apoptosis or a soluble protein that inhibits apoptosis [38].

**Figure 3:**
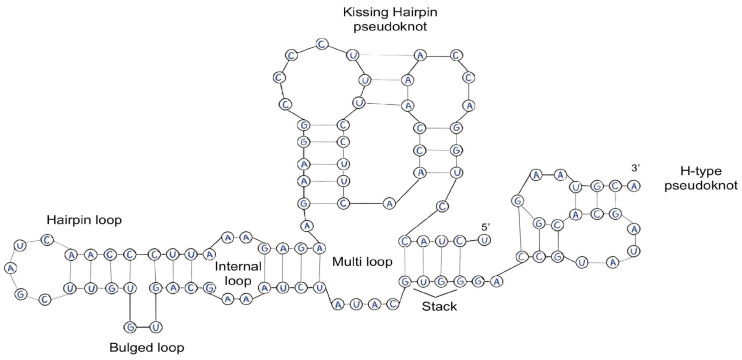
Possible variants of the secondary structure of RNA.

The secondary structure of RNA, arising through a variety of types of interactions, is an active and functionally relevant system that plays a key role in maintaining a wide range of biological processes (Figure 3) [39].

**Figure 4:**
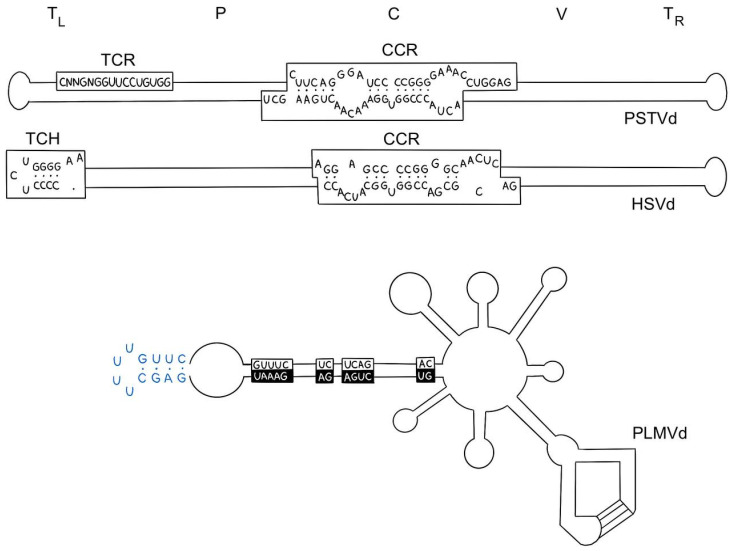
Structural features of viroids. The upper and middle schemes of the characteristic rod-shaped secondary structures of the genomic RNAs of the potato tuber fusiformity viroid (PSTVd) and hop dwarfism viroid (HSVd), respectively. The lower scheme corresponds to the multi-branched secondary structure of the hidden peach mosaic viroid genomic RNA (PLMVd) [45].

The stability of RNA’s secondary structure is a key factor determining its functional properties and interactions with other molecules [40]. Several features of RNA’s secondary structure significantly affect its stability:

1. Repeated sequences and palindromes in RNA are capable of forming different secondary structures, which can affect the accessibility of RNA to ribosomes and its resistance to degradation [41]. One example of the effect of palindromes on stability is viroids, a type of infectious agents that consist of a single RNA molecule. They do not have a protein coat and cannot replicate independently but can infect plants and animals [42,43]. Viroids contain a palindromic structural loop (Figure 4), which plays an important role in stabilizing the RNA and ensuring its function. This structure allows the viroid to multiply in the cell, infecting plants and animals. In addition, the palindromic structural loop may play a role in the regulation of gene expression in the cell, which can lead to changes in the cellular response to infection [44].

2. The concentration of complementary bases in the stroma also affects the stability of RNA. The higher the concentration of complementary bases, the more stable the RNA. The number of hydrogen bonds between complementary base pairs plays an important role in this. Guanine–cytosine (GC) pairs form three hydrogen bonds, and adenine–uracil (AU) pairs form two hydrogen bonds. The free energy associated with the hydrogen bonds in the guanine–cytosine (GC) pair is approximately −3.4 kcal/mol, which is significantly higher compared to the adenine–uracil (AU) pair, for which the energy is approximately −2.1 kcal/mol [46]. The actual binding energies may vary depending on the ionic strength of the medium, as well as the presence of proteins and other molecules that stabilize RNA. For example, Mg^2+^ cations can further stabilize GC-rich regions by shielding negative charges, further increasing the stability of these regions [47,48]. Thus, a higher proportion of GC pairs increases the thermodynamic stability of RNA [49]. It is important to note that, as in the case of the primary structure, the higher the binding energy between complementary bases, the more stable the RNA [50].
3. As has been noted earlier, the number and type of pseudoknots play a key role in modifying the thermodynamic stability of RNA molecules. Pseudoknots forming additional interbase bonds are able to minimize entropic changes in the system and increase the Gibbs free energy, which contributes to the overall stability of the molecule. These structural elements influence the prevention of RNA denaturation and degradation [51,52,53].
4. Methylation (m^6A, m^5C, Nm, m^7G described previously) and other post-transcriptional modifications, such as pseudouridylation (Ψ) and cytosine acetylation (ac4C), play critical roles in regulating the stability of RNA secondary structure [54,55]. The most common modification is m6A-type methylation (N6-methyladenine), which occurs both in the nucleus and in the cytoplasm [56]. This modification not only prevents RNA degradation under the influence of RNases but also increases the affinity of the molecule to specific protein factors involved in the regulation of genetic expression. Proteins binding to methylated RNA protect it from degradation by preventing its interaction with complexes responsible for degradation, such as exosomes [54].

**Figure 5:**
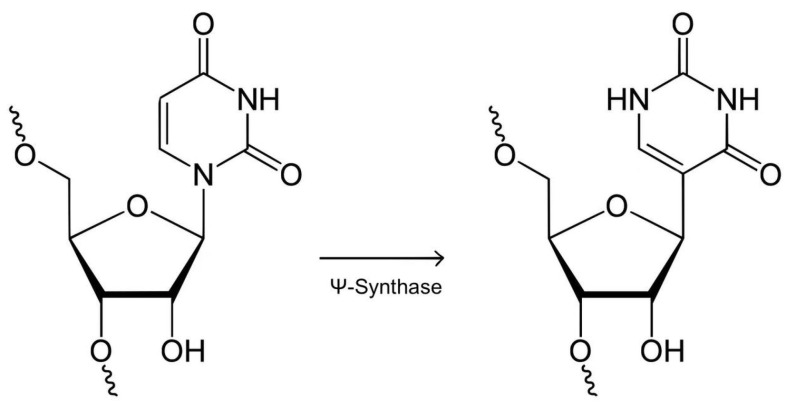
Scheme of RNA pseudouridylation (Ψ) catalyzed by pseudouridine synthase [61].

Pseudouridylation (Ψ) is involved in the regulation of gene expression and the stabilization of RNA molecules [57]. During this process, uracil undergoes isomerization from the trans-isomeric to the cis-isomeric form, which is carried out with the participation of specific enzymes, such as pseudouridine synthases [58]. The process of pseudouridylation is shown in Figure 5. 

The replacement of uracil with pseudouridine stabilizes the secondary structure of RNA due to additional hydrogen bonding between pseudouridine and ribose. This modification is often found in rRNA and tRNA modifications, indicating that they are properly stacked and functioning, and helps RNA resist the negative effects of the environment and cellular stress. Importantly, pseudouridylation in tRNA is important for the accuracy of transmission. The introduction of pseudouridylation into the anticodon loop of tRNA increases the flexibility and mobility of its structure, which promotes more precise pairing with mRNA codons in the ribosome. This modification allows the ribosomes to recognize the corresponding amino acid code more accurately, reduces the likelihood of errors in the translation process, and thus, allows for more correct protein synthesis. Pseudouridylation in tRNA provides high accuracy and efficiency in translation, which is especially important under conditions of rapid growth or stressful cellular processes [59,60].

Cytosine acetylation (ac4C) is a rarer but important modification catalyzed by the NAT10 enzyme. This modification is commonly found in mRNAs and tRNAs and plays a role in enhancing transcript stability and improving translation efficiency. The addition of an acetyl group to cytidine not only stabilizes mRNA structurally but also reduces its susceptibility to endonuclease degradation. Thus, ac4C protects the mRNA by increasing its lifespan and maintaining its availability for translation. In addition, cytosine acetylation increases the accuracy of ribosomal codon reading, which reduces error rates and promotes more efficient protein translation, especially under conditions of cellular stress, such as oxidative stress or temperature changes [62].

### 2.3. Features of the Tertiary Structure of RNA

The tertiary structure of RNA is a complex three-dimensional shape formed by interactions between nucleotide bases, the sugar–phosphate chain, and metal ions. The tertiary structure is also represented by trunks, loops, and quadruplex groups of four bases that interact with each other depending on their type. It is important to note that, in addition to the above-described structural features of the RNA molecule**,** metal ions play an important role in stabilizing and destabilizing the tertiary structure. Studies have shown that Mg^2+^, Ca^2+^, and Mn^2+^ ions have a stabilizing effect on the RNA structure by interacting with its phosphate groups and nitrogenous bases and participating in the catalysis of splicing reactions [40]. 

For example, zinc ions (Zn^2+^) can selectively bind to certain amino acids in the C-terminal region of the ACE2 receptor, which significantly affects its interaction with the S-protein of the SARS-CoV-2 virus, changing the level of viral infectivity [63]. At the same time, copper ions (Cu^2+^) exhibit a dual nature of the effect on RNA: they can both induce denaturation and stabilize specific secondary and tertiary RNA structures [64]. The sources of metal ions in the cell are diverse. The main ones are ions from the environment, including magnesium (Mg^2+^), manganese (Mn^2+^), iron (Fe^2+^/Fe^3+^), and zinc (Zn^2+^). These ions can be captured by RNA from the surrounding solution, leading to the formation of metal–RNA complexes that contribute to its stabilization. Moreover, metals play an important role in enzymatic reactions: enzymes involved in RNA biosynthesis, such as RNA polymerase, contain metal ions in their active centers. RNA polymerase needs the presence of magnesium or manganese ions to successfully carry out the RNA synthesis process, and these ions can be incorporated into the newly synthesized RNA molecule. In addition to RNA polymerases, RNase H (Ribonuclease H) uses magnesium to cleave RNA in RNA-DNA hybrid molecules [63], and RNase P (Ribonuclease P) uses magnesium to process tRNA precursors [65]. The spliceosome (complex ribonucleoprotein complex), which catalyzes the excision of introns from pre-mRNA, also depends on magnesium ions to stabilize its temporary active center [66].

The structural stability of RNA is due not only to coordination with metal ions but also to hydrophobic interactions between nitrogenous bases and the sugar–phosphate backbone [67].

The additional structural elements of RNA, such as quadruplex and pseudoknots, play a significant role in the stabilization of RNA molecules. G-quadruplex in the 5′-untranslated regions (5′-UTR) of mRNA can influence the translation process by the binding of ribosomes or regulatory proteins [68]. In addition, G-quadruplexes are found in rRNAs and are involved in stabilizing the structure of the ribosome, which is critical for its function [67].

U-tetrads are a structural element found in the G-quadruplexes of RNA. They are structures formed by uracil-rich sequences, a structure of four uracil molecules linked by hydrogen bonds that further stabilize the secondary structure of RNA [69]. U-turns in the case of miRNAs play an important role in stem-loops, where they help stabilize the secondary structure and improve the interaction with target mRNAs [70,71].

## 3. External Factors Affecting RNA Stability

The external factors affecting RNA stability can be divided into several categories, which are summarized in Table 1. 

Thus, RNA, being highly chemically unstable, is easily subject to degradation and changes under the influence of various external factors. Even minor changes in the environment can significantly affect its functional role within the cell.

## 4. A Modern Solution to the Problem of RNA Stabilization

In recent years, a whole series of techniques have been developed to stabilize RNA and prevent its degradation. Modern methods of RNA stabilization include the following:

1. RNA-stabilizing reagents effectively inhibit the activity of RNases, preventing the enzymatic degradation of RNA molecules. The actions of such reagents can vary: some bind to the active center of RNases, blocking their access to RNA, while others change the structure of the enzyme or substrate, making interaction between them impossible. For example, some of the most popular reagents that inhibit RNase activity are RNasin, dithiothreitol, β-mercaptoethanol, vanadyl ribonucleoside complexes, and aurintricarboxylic acid, which binds to the active center of nucleases, prevents their activity, and protects nucleic acids from degradation. In addition, it neutralizes free radicals, protecting cells from oxidative stress, which helps stabilize RNA. RiboLock works differently: the reagent changes the structure of RNases, which prevents them from interacting with RNA. It is important to note that, despite the existing positive effects and advantages of RNA-stabilizing reagents, they can inhibit the activity of enzymes other than RNases, be toxic to cells or organisms, and have a high cost [112,113,114]. 

It is important to mention diethylpyrocarbonate (DEPC) [115,116,117], which is widely used in molecular biology to inactivate ribonucleases (RNases). DEPC covalently modifies the predominantly imidazole group of histidine to form carbethoxylated derivatives [118]. This modification disrupts key functions of the active center of RNases and the ability of the enzymes to catalyze the hydrolysis of phosphodiester bonds in RNA. DEPC-treated solutions play a key role when working with RNA, including miRNA, where even minimal RNase activity can skew the results of the assay. DEPC effectively prevents RNA degradation during RNA isolation, storage, and analysis [115,116], but its use is limited in solutions containing primary amines or Tris-buffer, with which it reacts and loses activity [116,117,119].

2. RNA-stabilizing matrices are special materials used to stabilize and store RNA in tissue and cell samples. They are three-dimensional structures that can be made of various materials, such as silicone, polyethylene, polyester, etc. The matrix absorbs RNA from the sample and stabilizes its structure, preventing degradation and inactivation. The matrix chelates metal ions that can activate ribonucleases and inactivate RNA. Examples of RNA-stabilizing matrices are the commercial systems RNAgard (Epicentre) and Stabilizor (Ambion), which are polyethylene and silicone matrices, respectively, that stabilize the RNA structure and inhibit ribonuclease activity. The main disadvantages of using matrices are their high cost, the need for additional equipment for storage and transport, and the presence of restrictions in use for certain types of samples [120,121].

3. Cryopreservation is a method of storing RNA at temperatures typically below −80 °C to prevent RNA degradation, structure stabilization, and inactivation. Current approaches to RNA cryopreservation represent an important tool for the long-term storage of labile biomaterial in a stable state. This method is particularly valuable for a multitude of applications including genetic disease research, the development of advanced diagnostic technologies, and innovative therapeutic strategies. Storing RNA in liquid nitrogen at extremely low temperatures, as low as −196 °C, ensures its exceptional stability. However, this approach requires specialized equipment and incurs high financial costs. An alternative method is to store RNA on dry ice at −80 °C, which, while more economically accessible, has certain limitations for specific sample types [122].

4. Lyophilization, as a method of RNA preservation by removing water, makes it possible to ensure the long-term preservation of the molecule in a stable state. Under dry conditions, the structure of RNA is stabilized, which prevents its denaturation and degradation, and the absence of water significantly reduces the activity of ribonucleases [123,124,125]. However, lyophilization requires special equipment and additional procedures to recover RNA prior to analysis, which adds complexity and cost to the process [126]. Commercial systems, such as QIAamp (Hilden, Germany, Qiagen), PureLink (Waltham, MA, USA, Thermo Fisher Scientific), and RNAqueous (Waltham, MA, USA, Thermo Fisher Scientific) offer solutions for RNA purification, not specifically lyophilization; although, some kits may be used in conjunction with lyophilized samples. The main disadvantage of lyophilization as a method of RNA preservation is low RNA recovery, because, after lyophilization, the molecule becomes more prone to aggregation (clusters of molecules) due to an increase in nonspecific intermolecular interactions [127]. Aggregation makes the molecules less soluble in water upon redissolution. To increase the yield of RNA, additional redissolution manipulations are applied using buffers containing disaggregating agents. For example, 1 mM EDTA helps prevent aggregate formation by binding ions that may contribute to these interactions [128]. Detergents, such as Tween-20 or Triton X-100, reduce surface tension and promote the dispersion of RNA molecules, improving solubility [129].

5. The use of RNA-stabilizing nanoparticles, such as gold and lipid nanoparticles, open new horizons in protecting RNA from degradation, including the inhibition of ribonuclease activity and the prevention of oxidative stress. Lipid nanoparticles (LNPs) composed of lipids are widely used to deliver RNA into cells, while polymeric nanoparticles (PNPs) and metal nanoparticles (MNPs) provide additional protection for the molecule. Despite the significant advantages, nanoparticles can induce immune reactions and require additional RNA purification steps, which also increases the complexity and cost of the procedures [130]. 

Importantly, recent advances in RNA nanotechnology have focused on improving the stability of RNA for therapeutic applications, including RNA interference (RNAi). Leading research from Peixuan Guo’s laboratory at The Ohio State University is focused on creating multifunctional RNA nanoparticles that are resistant to the action of ribonucleases. Specifically, the developed delivery systems based on “helical motor” RNA complexes provide effective protection for therapeutic small interfering RNAs (siRNAs) and allow them to be delivered to target cells with high efficiency. These structures increase the stability of RNA and its therapeutic effect in cellular systems, which makes them promising for applications in oncology and other medical fields [131,132].

6. RNA stabilization technologies based on interaction with nucleic acids are innovative strategies to protect RNA from degradation and inactivation, which are based on the ability of nucleic acids to form specific and stable complexes with target RNA. This stabilization process is due to the formation of duplexes between RNA and complementary nucleic acids, which significantly increases the resistance of RNA to exogenous and endogenous influences. Among the key approaches that have found application in this field are RNA–RNA duplexes [133,134] or RNA–DNA duplexes [135], aptamers—short oligonucleotides—with a high affinity to the target RNA and are capable of forming stable complexes [136], nanoclusters [137], and hybrid oligonucleotides [138]. 

One of the key directions in this field is the creation of dynamic RNA nanostructures, which can form complex assembled structures that are resistant to degradation under biological conditions. These nanostructures are designed for targeted delivery of small interfering RNA (siRNA), microRNA (miRNA), and other therapeutic molecules [139]. Kirill Afonin and colleagues, using computer modeling, have designed three-dimensional cubic RNA structures that are assembled from multiple RNA strands. These structures, assembled from short RNA segments, are highly stable and can retain their functionality for long periods of time, opening new perspectives for medical applications [140].

7. Stabilizing buffers are solutions that are used to stabilize and protect RNA from degradation during storage, transport, and manipulation. The buffers include various chemical reagents that block the activity of ribonucleases. The composition of such buffers is often kept confidential and protected by trade secrets. Among the most popular commercially available RNA-stabilizing buffers are RNAlater (Waltham, MA, USA, Thermo Fisher Scientific) [141] and RNAprotect (Hilden, Germany, Qiagen) [142], which inhibit ribonuclease activity, denature proteins, and bind metal ions. However, stabilizing buffers have disadvantages: some components, such as SDS, can interfere with further steps of RNA analysis, such as reverse transcription or PCR, by inhibiting enzyme activity. Many buffers do not ensure RNA stability over long periods of time (2 weeks or more) over a wide range of temperatures [143]. Buffers can also affect the quality of the RNA structure, and their use may require additional steps, such as the purification of the RNA from the buffer [144,145], which can be time- and resource-intensive. In addition, RNA-stabilizing buffers have a high cost. 

Nevertheless, it should be noted that these methods are associated with certain limitations, including the need for additional purification steps and difficulties in adapting the technologies for specific RNA types, which potentially limit their practical applicability.

8. The chemical modifications of ribose have become an important area for increasing the stability and functionality of RNA molecules used in biomedicine [99,146]. The 2′-O-methylation of ribose is the addition of a methyl group to the 2′-hydroxyl group of ribose in a nucleotide. This change makes RNA less susceptible to enzymatic hydrolysis, preventing the molecule from being broken down by ribonucleases. Nucleotides that are 2′-O-methylated effectively block interaction with enzymes, increasing the stability of RNA structures in biological media and prolonging the time of their activity [146,147].

Ribose fluorination is a chemical modification that places fluorine in the 2′ ribose position (replacing the OH of the natural ribose). This modification makes RNA less susceptible to enzymatic degradation and hydrolysis, allowing it to be used for long-term storage and therapeutic applications, such as RNAi and mRNA vaccines [148]. The fluorine atom at the 2′ position of the ribose has a stable electronic effect, affecting the conformation of the ribose and the entire RNA strand [149]. This makes fluorinated RNA less reactive, which protects it from hydrolysis and improves its physicochemical stability. It helps maintain its structure in biological environments where unmodified RNA is rapidly degraded.

9. RNA stabilization by RNA chaperones, such as Hfq, ProQ, and RrpA, opens new perspectives. These chaperones interact with RNA to form stable complexes that protect it from denaturation and inhibit ribonuclease activity. As a result, the stability and functional activity of RNA is increased. Nevertheless, this method is fraught with certain difficulties. Firstly, competition with other proteins for binding to RNA may reduce the efficiency of chaperones. Secondly, there is a risk of nonspecific binding to other RNAs, which may cause undesirable side effects. Additionally, chaperones are susceptible to degradation and inactivation within the cell, further limiting their application. The cost of producing the highly purified proteins required to realize these technologies remains a significant obstacle, especially in the context of working with microRNAs and small interfering RNAs [150,151].

## 5. Key Recommendations for RNA Storage 

Based on the data described in this article, we have formulated key recommendations for RNA storage aimed at minimizing degradation and damage to the molecule:

1. For long-term storage of RNA, temperatures of −80 °C or below are optimal. It is recommended to use reliable ultracoolers or liquid nitrogen storage containers to ensure minimal loss of stability of the molecule.
2. An important factor is the use of tubes specifically designed for RNA storage. They should be airtight and watertight, which significantly reduces the risk of contamination and prevents degradation of the molecules.
3. Avoid freeze–thaw cycles, as repeated freezing cycles can cause RNA degradation and fragmentation. To avoid this, it is advisable to use aliquots.
4. Use DEPC (diethyl pyrocarbonate). It is recommended to use DEPC-treated water for the preparation of all buffers and solutions. Typically, 0.1% DEPC is added to the water, then incubated at room temperature (about 20–25 °C) for 12–18 h followed by autoclaving to remove residual reagent. DEPC irreversibly inactivates ribonucleases by modifying their active centers during incubation, which prevents RNA degradation [152]. This simple and economical method significantly increases the stability of RNA storage.
5. For RNA storage, it is recommended to use buffers, such as TE buffers or the previously described commercial stabilization solutions. It is important to consider the composition of the solutions depending on the subject and the objectives of the study. For example, the presence of EDTA in the buffer may bind metal ions, which negatively affects enzymatic processes in subsequent studies. However, EDTA can also be beneficial by inhibiting RNase activity and preventing the formation of secondary structures.
6. When working with RNA, only sterile laboratory equipment, consumables, and reagents should be used to minimize the risk of degradation of the molecule by RNases.
7. RNA should be stored in complete darkness, as exposure to light can provoke photochemical reactions leading to degradation.

The chemical structure of RNA makes it vulnerable to hydrolysis, oxidation, and enzymatic degradation. These features contribute to its easy degradation during storage and analysis, which in turn can lead to erroneous results. Following the above recommendations will help to ensure the integrity of RNA samples during storage.

## 6. Conclusions

The study of RNA stabilization is a challenging area in modern molecular biology and is still ongoing. Understanding the mechanisms of RNA stabilization can help to develop new strategies to conserve RNA and study its functions. In this review, we have addressed the complex interactions between intrinsic and extrinsic factors affecting RNA stability, including interactions with proteins, specific chemical modifications, and the influence of environmental conditions. This review emphasizes the importance of an in-depth study of the molecular processes that determine RNA stability and discusses the current approaches to address this challenge. In the future, solving the problem of RNA instability may help to achieve a number of important goals in medicine and biotechnology. For example, in the development of effective vaccines against viral infections, as well as in the creation of biobanks and new methods for the treatment of cancer. 

## Figures and Tables

**Table 1:** Factors and environment affecting the stability and functionality of RNA.

Categories	Factor	Effect
Physical	Temperature	Increased temperature accelerates RNA hydrolysis. Conversely, low temperatures enhance RNA stability by reducing molecular energy and strengthening weak interactions [72]. However, some viral RNAs can denature outside a specific temperature range (e.g., <0 °C or >50–60 °C) [73]. Optimal storage: −20 °C to −80 °C [74].
	pH	A slightly acidic pH (5.5–6.5) is often used for in vitro storage, but there is no single “optimal” pH for all types of RNA [75]. At low pH values, RNA undergoes hydrolysis. Hydrogen bond breaking occurs in the presence of chaotropic agents, such as urea, lithium oxide, trifluoroethanol (TFE), or guanidinium salts [11,76]. At high pH values, RNA undergoes alkaline hydrolysis rather than hydrogen bond breaking as previously stated. In this process, hydroxide ions attack phosphodiester bonds, resulting in chain breakage and RNA degradation [11,77]. RNA can undergo chemical modifications, such as oxidation and deamination, at extreme pH values [78,79,80].
	Relative humidity	High relative humidity (>50%) promotes hydrolysis and RNase activity, leading to degradation. Moderate relative humidity (20–50%) increases susceptibility to hydrolysis and denaturation. Low relative humidity (<20%) enhances stability but may increase oxidation risk. Store RNA in airtight containers with desiccant under low humidity conditions [81,82].
	Ionic strength	Optimal ionic strength varies (10 mM to several hundred mM) depending on RNA characteristics (type, length, sequence, structure) and experimental conditions (e.g., temperature, pH) [75,83]. Increasing the strength of the ionic solution (using Mg^2+^, Na^+^) helps stabilize the secondary and tertiary structure of RNA due to electrostatic repulsion between phosphate bonds [48]. Low ionic strength of the solution promotes degradation and increases RNase activity and the destabilization of the RNA structure, leading to denaturation and loss of function [75,83].
Chemical	Reactive oxygen species (ROS)	The oxidation of RNA bases leads to the formation of mutations and disruption of the molecule’s function. The oxidation of phosphodiester bonds leads to RNA strand breaks and RNA fragmentation [3,84,85]. The modification of RNA polymerase by ROS leads to changes in transcription and gene expression [3,84].
	Reactive nitrogen species (RNS)	The nitrosation of guanine and cytosine by nitric oxide (NO•) leads to the formation of modified forms, such as 8-nitroguanine and nitrosated cytosine. The formation of 8-nitroguanine and other nitrosyl derivatives makes RNA more susceptible to breakage and degradation. In addition, nitrosation can disrupt the proper stacking of RNA, reducing its structural integrity and affecting its ability to interact with proteins, ribosomes, and other molecules [86,87]. The oxidation of phosphodiester bonds by peroxynitrite (ON-O-O^−^) leads to RNA chain breaks (especially in loops and hairpins). These breaks reduce the stability of the RNA, leading to its degradation and reduced functionality in the cell [88,89]. RNS can interact with the amino acid residues of RNA polymerase, causing their nitrosation and oxidation. Damage to RNA polymerase leads to incorrect transcript length or RNA synthesis, which reduces its resistance to nucleases and accelerates its degradation [88,90,91,92].
	Metal ions	Metals that stabilize RNA: - Mg^2+^ coordinates with RNA, neutralizing phosphate charges and stabilizing its structure [93,94,95]; - Mn^2+^ stabilizes RNA structure and acts as a cofactor for RNA polymerase [94,95]; - Zn^2+^ stabilizes specific RNA structures and can modulate RNA polymerase activity [94,95,96]. Metals that destabilize RNA: Cu^2+^, Fe^2+^, and Co^2+^ catalyze RNA degradation via oxidative stress (AOS) and hydrolysis of phosphodiester bonds [94,95,97].
	Chemical reagents	Organic solvents, such as dimethyl sulfoxide (DMSO), protect RNA from degradation, particularly during freezing and thawing [98]. Chemical modifications (e.g., Ψ) enhance resistance to ribonucleases and unfavorable external factors [99]. The effect of other modifications (e.g., m^6A, m^5C, Nm, m^7G, m^6Am, ac4C) on RNA stability is complex and context-dependent [99,100,101]. Denaturing agents, such as trifluoroethanol (TFE) or guanidinium salts, destabilize RNA secondary structure [76,102].
Biological	Enzymes	Degrading: - RNases (e.g., RNase A, H, L)—break down RNA molecules [6,40]; - RNA exonucleases—degrade RNA from either the 3′ or 5′ end [6]. Modifying: - RNA ligases (e.g., T4 RNA ligase)—catalyzes the formation of covalent bonds between RNA molecules [40]; - RNA editases (e.g., ADAR)—chemically modify RNA bases, such as converting adenosine to inosine [103]; - RNA methyltransferases (e.g., RNMT1)—add methyl groups to specific nucleotides in RNA [40]; - RNA demethylases (e.g., FTO)—remove methyl groups from modified nucleotides in RNA [40]; - RNA uridyltransferases (e.g., TUTase)—add uridine residues to the 3′ end of RNA [104]. Synthesizing: - RNA polymerases (e.g., RNA polymerase I, II, and III in eukaryotes)—synthesize RNA molecules from a DNA template [40].
Mechanical	Mechanical impact	Mechanical stress (e.g., intense pipetting, shaking, high-speed centrifugation) can fragment RNA, increasing degradation [105,106].
	Ultrasound	Ultrasound generates cavitation, localized high pressure/temperature, and free radicals [92], leading to RNA degradation (e.g., strand breaks, oxidation) and structural changes [107].
Radiation	Ionizing radiation	Ionizing radiation can induce RNA damage through the generation of free radicals, leading to oxidative damage and strand breaks [108,109]. This can also cause conformational changes and denaturation [108,110].
	Ultraviolet radiation	UV radiation leads to denaturation, photochemical reactions, free radical generation, and the formation of cyclobutene pyrimidine dimers [111], impairing RNA structure and function [80].
